# Supplementary material for: Genome-wide identification and analysis of ascorbate peroxidase (APX) gene family in hemp (Cannabis sativa L.) under various abiotic stresses
Source: PeerJ. 2024 Apr 26;12:e17249. doi: 10.7717/peerj.17249 (PMC11057428; doi:10.7717/peerj.17249)
Supplement: Table S1 [file peerj-12-17249-s001.docx]

| **Gene name** | **Forward primer(5’–3’)** | **Reverse primer(5’–3’)** |
| --- | --- | --- |
| CsAPX1 | TCCAACTATCGACTTTGTCTACGG | GGTCTTTTGTCCATGCTCCTTC |
| CsAPX2 | AAGTCCAAACATCCGAAGATTACC | CCACCAGAAAGTGCCACAATG |
| CsAPX3 | CTGGTGTTGTTGCAGTCGAG | CCTTATCCGACAGGCCCATT |
| CsAPX4 | TGACCCGTCTTTCAGAGCGT | CAGCTCTCTCTTGCCAGTCG |
| CsAPX5 | AAGGACCCTGGACAAACAACC | AGCCTCAGCGTAATCAGAAAAGA |
| CsAPX6 | TTCCTCCGCTCACCCTCTTC | AGCCCTGCCCTTGGGAACTAC |
| CsAPX7 | TTTGATGTGAAAACCAAGACCG | GCGTAAGAAAGAATGGGGAACTG |
| CsAPX8 | AGCAGAATCACGATGCCAACAC | AACAGCAACTACTCCAGCCAACT |
| Actin | TTGCTGGTCGTGATCTTACTG | GTCTCCATCTCCTGCTCAAAG |
