# Supplementary material for: Genome-wide identification and analysis of ascorbate peroxidase (APX) gene family in hemp (Cannabis sativa L.) under various abiotic stresses
Source: PeerJ. 2024 Apr 26;12:e17249. doi: 10.7717/peerj.17249 (PMC11057428; doi:10.7717/peerj.17249)
Supplement: Supplemental Information 3 [file peerj-12-17249-s003.doc]

EXPERIMENTAL DESIGN:

1. Definition of experimental and control groups

The experimental groups were hemp seedlings that were subjected to four adversity stresses at different times. Control groups are hemp seedlings not involved in any treatment.

1. Number within each group

Approximately 100mg of leaves

SAMPLE:

1. Description

Leaf samples were collected at 0, 3, 6, 12, 24, and 48 hours after four treatments, with approximately 100 mg of leaves collected at each time point.

1. Microdissection or macrodissection

Macro

1. Processing procedure

The study utilized the hemp cultivar “Qingma 3.” For the expression analysis of *CsAPXs* under cold stress, seeds were planted in a mixture of peat moss and perlite in a nutrient bowl with a ratio of 3:1. And for the expression analysis under drought, salt, and oxidative stresses, hemp were cultivated in a hydroponic box. These plants were cultivated in a growth chamber under controlled conditions with a day/night temperature regimen of 24 ℃/16 ℃ and a photoperiod of 16 h of light and 8 h of darkness. For cold stress treatment, four-week-old seedlings cultured in growth chambers were placed in the 4 °C climate chamber. For salt, oxidative, and drought stress treatment, the seedlings were treated with Hoagland’s nutrient solution enriched with 250 mmol/L NaCl, 10 mmol/L H_2_O_2_, and 20% PEG6000. Leaf samples were collected at 0, 3, 6, 12, 24, and 48 hours after treatment, with approximately 100 mg of leaves collected at each time point. All of the treatments were performed with three biological replications. Subsequently, all samples were promptly frozen in liquid nitrogen and stored at -80°C for RNA extraction.

1. If frozen - how and how quickly?

The leaves were wrapped in tinfoil and frozen in liquid nitrogen, then transferred to a -80°C refrigerator.

1. If fixed - with what, how quickly?

No fixed

1. Sample storage conditions and duration (especially for FFPE samples)

-80°C and within 24 hours

NUCLEIC ACID EXTRACTION

1. Procedure and/or instrumentation

Total RNA was extracted from hemp plant samples employing the Trizol method.

1. Name of kit and details of any modifications

Total and no details of any modifications

1. Details of DNase or RNAse treatment

RNase-free material was used for all extractions. Removal of genomic DNA using DNase scavengers.

1. Contamination assessment (DNA or RNA)

Assessing contamination using a spectrophotometer.

1. Nucleic acid quantification

Samples with OD260/280 between 1.9 and 2.1 have better purity.

1. Instrument and method

OD measurement by spectrophotometer.

1. RNA integrity method/instrument

RNA electrophoresis. Agarose gel electrophoresis.

1. RIN/RQI or Cq of 3' and 5' transcripts

RIN/RQI or Cq of 3' and 5' transcripts are all within the normal range.

1. Inhibition testing (Cq dilutions, spike or other)

No inhibitiob testing.

REVERSE TRANSCRIPTION

1. Complete reaction conditions

For best results, store the kit at -20 °C. Avoid storing or leaving at 4 °C or room temperature.

Gently invert the centrifuge tube several times to mix the reagents and avoid foaming, centrifuge briefly and prepare for use.

Follow the procedure to avoid RNase contamination.

Prepare all reactions on ice to avoid RNA degradation.

1. Amount of RNA and reaction volume

Use purified poly A RNA in amounts ranging from 1 ng ~ 100 ng. The total volume of the reaction was 20 μl.

1. Priming oligonucleotide (if using GSP) and concentration

No using GSP

1. Reverse transcriptase and concentration

SureScript RTase Mix (20×). The final concentration is 1×.

1. Temperature and time

25℃ 5min, 42℃ 15min, 85℃ 5min, 4℃ hold.

qPCR TARGET INFORMATION

1. If multiplex, efficiency and LOD of each assay

It's not multiplex.

1. Sequence accession number

| *CsAPX1* | XP_030485953.1 |
| --- | --- |
| *CsAPX2* | XP_030485951.1 |
| *CsAPX3* | XP_030487766.1 |
| *CsAPX4* | XP_030491786.1 |
| *CsAPX5* | XP_030493443.1 |
| *CsAPX6* | XP_030496013.1 |
| *CsAPX7* | XP_030504263.1 |
| *CsAPX8* | XP_030490534.1 |

1. Amplicon length

The amplification lengths of all eight genes ranged from 150 to 300 bp.

1. In silico specificity screen (BLAST, etc)

Blast comparisons of primer specificity have been performed in the NCBI.

1. Location of each primer by exon or intron (if applicable)

All primers are on exons.

1. What splice variants are targeted?

Target has no shear variants.

qPCR OLIGONUCLEOTIDES

1. Primer sequences

| Gene name | Forward primer (5’–3’) | Reverse primer (5’–3’) |
| --- | --- | --- |
| CsAPX1 | TCCAACTATCGACTTTGTCTACGG | GGTCTTTTGTCCATGCTCCTTC |
| CsAPX2 | AAGTCCAAACATCCGAAGATTACC | CCACCAGAAAGTGCCACAATG |
| CsAPX3 | CTGGTGTTGTTGCAGTCGAG | CCTTATCCGACAGGCCCATT |
| CsAPX4 | TGACCCGTCTTTCAGAGCGT | CAGCTCTCTCTTGCCAGTCG |
| CsAPX5 | AAGGACCCTGGACAAACAACC | AGCCTCAGCGTAATCAGAAAAGA |
| CsAPX6 | TTCCTCCGCTCACCCTCTTC | AGCCCTGCCCTTGGGAACTAC |
| CsAPX7 | TTTGATGTGAAAACCAAGACCG | GCGTAAGAAAGAATGGGGAACTG |
| CsAPX8 | AGCAGAATCACGATGCCAACAC | AACAGCAACTACTCCAGCCAACT |

1. Location and identity of any modifications

Location and identity without any modifications.

qPCR PROTOCOL

1. Complete reaction conditions

Performance-enhanced antibody-mediated Taq DNA polymerase.

Unique reference dye is compatible with all qPCR instruments.

Optimized buffer enhances specificity and reduces primer-dimer formation.

High sensitivity, specificity and reliability.

1. Reaction volume and amount of cDNA/DNA

The total volume of the reaction is 10 μL. The amount of cDNA is 1.0 μL.

1. Primer, (probe), Mg++ and dNTP concentrations

The concentration of PCR forward primer and PCR reverse primer are all 0.2 μM.

1. Polymerase identity and concentration

5×BlazeTaq Qpcr Mix^a^. The concentration of 5×BlazeTaq Qpcr Mix^a^ is 1×.

1. Buffer/kit identity and manufacturer

The kit is BlazeTaq™ SYBR Green qPCR mix 2.0. The BlazeTaq^TM^ SYBR Green qPCR Mix 2.0 reagent from GeneCopoeia Company was used.

1. Additives (SYBR Green I, DMSO, etc.)

There's only one enzyme, BlazeTaq Qpcr Mix^a^.

1. Complete thermocycling parameters

Detailed procedures: Stage 1, 95 ℃ 30 s for 1 cycle; Stage 2, 95 ℃ 10 s, 60 ℃ 30 s, 65 ℃ 10 s for 40 cycles.

1. Manufacturer of qPCR instrument

The qRT-PCR analysis was performed using a BIO-RAD CFX90 quantitative PCR instrument.

qPCR VALIDATION

1. Specificity (gel, sequence, melt, or digest)

The amplification products of each gene were specific.

1. For SYBR Green I, Cq of the NTC

Cq of the NTC is 0.000. NTC is not amplified.

1. PCR efficiency calculated from slope

The slopes are all in the range of -3.3 to -3.6. The PCR efficiency are 0.895 - 0.968.

1. r2 of standard curve

All standard curves had r2 greater than 0.98.

1. Linear dynamic range

The dynamic range included 3 orders of magnitude. Each set of experiments approached 5 to 6 Log10 concentrations.

1. Cq variation at lower limit

Lower limit Cq varies within reasonable limits.

1. Evidence for limit of detection

The limit of detection for all experimental groups were close to 3.

1. If multiplex, efficiency and LOD of each assay.

No multiplex.
